# Salivary Cortisone as a Potential Alternative to Cortisol in Periodontitis Severity Assessment

**DOI:** 10.3390/ijms27020805

**Published:** 2026-01-13

**Authors:** Dimitar Dimitrov, Antoaneta Mlachkova, Velitchka Dosseva-Panova

**Affiliations:** Department of Periodontology, Faculty of Dental Medicine, Medical University of Sofia, 1431 Sofia, Bulgaria

**Keywords:** salivary cortisone, cortisol, periodontitis, glucocorticoids, stress, inflammation

## Abstract

Salivary cortisol is widely used to investigate stress–periodontitis interactions, but its measurement is affected by methodological limitations. Cortisone, the predominant salivary glucocorticoid, may offer analytical advantages, yet its role in periodontitis remains unexplored. This study evaluated salivary cortisone in relation to periodontal disease severity and compared its performance with cortisol. Sixty-seven periodontitis patients were classified as Stage I/II (*n* = 32) or Stage III/IV (*n* = 35). A comprehensive periodontal examination was performed, including FMPS, FMBS, PPD, CAL, BoP, and the BL/Age ratio. Unstimulated morning saliva samples were analyzed for cortisone and cortisol using liquid chromatography–tandem mass spectrometry, and for IL-1β and IL-6 using ELISA. Both cortisone and cortisol levels were significantly higher in Stage III/IV periodontitis (*p* = 0.014). Cortisone correlated strongly with cortisol (ρ = 0.523, *p* < 0.001) and was positively associated with IL-6 (ρ = 0.322, *p* = 0.008) and multiple clinical indicators of periodontal disease severity. ROC analysis showed comparable discriminatory performance for cortisone and cortisol (AUC = 0.675), with cortisone demonstrating higher specificity (94%) for Stage III/IV periodontitis. Our findings suggest that salivary cortisone performs similarly to cortisol and warrants further investigation as an alternative salivary glucocorticoid marker in periodontal research.

## 1. Introduction

Periodontitis is a prevalent, biofilm-induced inflammatory disease characterized by dysregulated host–microbial interactions that lead to progressive destruction of periodontal tissues. Beyond local microbial challenge, numerous systemic and behavioral factors modulate disease susceptibility and severity. Among these, psychological stress has gained increasing attention due to its effects on immune function, systemic inflammation, and disease progression through activation of the hypothalamic–pituitary–adrenal (HPA) axis. Elevated glucocorticoid output is a hallmark of HPA-axis activation, and accumulating evidence indicates that stressed individuals exhibit more severe periodontal breakdown and poorer treatment outcomes [1].

Clinical studies support a relationship between HPA-axis activity and periodontal disease severity [2,3,4]. Salivary cortisol concentrations are consistently higher in patients with periodontitis compared with periodontally healthy individuals. A recent meta-analysis demonstrated elevated cortisol levels particularly among stressed periodontitis patients [5]. Additional case–control studies have shown stepwise increases in salivary cortisol across disease stages [6] and associations between cortisol and clinical parameters of periodontal destruction, including probing pocket depth (PPD), clinical attachment loss (CAL), bleeding on probing (BoP), plaque accumulation, and the presence of *Porphyromonas gingivalis* [7]. For this reason, salivary cortisol has become a frequently used biomarker in studies examining the interaction between stress and periodontal inflammation.

**Figure 1:**
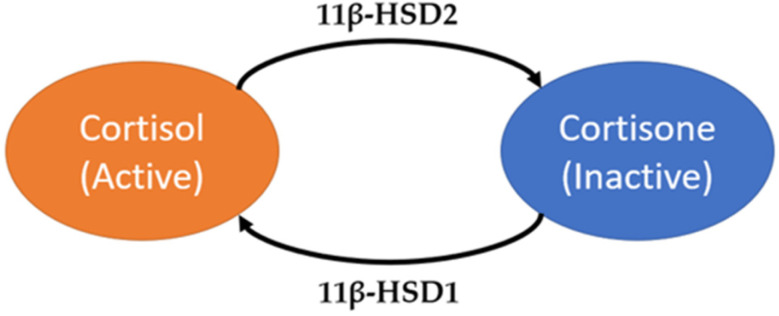
Conversion between cortisol and cortisone mediated by 11β-hydroxysteroid dehydrogenase enzymes.

However, interpretation of salivary cortisol data is constrained by methodological challenges. Most periodontal studies have relied on immunoassays, which are affected by cross-reactivity, limited specificity at low concentrations, and susceptibility to contamination. These limitations contribute to considerable heterogeneity in reported cortisol findings [5,6,7]. In addition, contemporary evidence indicates that local glucocorticoid metabolism is altered in periodontal tissues. Increased expression of 11β-hydroxysteroid dehydrogenase type 1 (11β-HSD1), the enzyme converting cortisone to cortisol (Figure 1), has been demonstrated in gingival tissues from patients with chronic periodontitis and in experimental inflammatory models [8,9,10,11]. These findings highlight the biological relevance of glucocorticoid pathways in periodontal inflammation.

In saliva, cortisol is largely converted to cortisone by 11β-HSD2 in the salivary glands, making cortisone the predominant glucocorticoid metabolite in oral fluid [12]. Recent endocrine studies using liquid chromatography–tandem mass spectrometry (LC–MS/MS) have shown that salivary cortisone provides a more stable and accurate estimate of free cortisol exposure than salivary cortisol. Cortisone demonstrates high agreement with circulating serum free cortisol, reliable detection even at low concentrations, and suitability for single-sample assessment [13,14,15,16,17,18]. Despite these advantages, salivary cortisone has not been investigated in periodontal disease, and no studies have directly compared cortisol and cortisone using LC–MS/MS in this context.

Given the biological relevance of glucocorticoid metabolism in periodontal inflammation, and the recognized physiological advantages of cortisone, examining salivary cortisone in periodontitis represents an important and unexplored area of investigation. The aim of this study was to evaluate salivary cortisone as a biomarker in patients with periodontitis and determine whether it performs as a reliable alternative to cortisol. We hypothesized that salivary cortisone, measured by LC–MS/MS, would demonstrate equal or superior ability to distinguish periodontal disease severity and correlate with inflammatory and clinical periodontal parameters compared with salivary cortisol.

## 2. Results

A total of 67 individuals participated in the study, with 32 classified as Stage I/II and 35 as Stage III/IV periodontitis. The mean age of the cohort was 51.4 ± 9.4 years, and the sample included 35 females and 32 males.

### 2.1. Comparison of Cortisone, Cortisol, and Cortisol/Cortisone Ratio Between Periodontitis Stages

**Figure 2:**
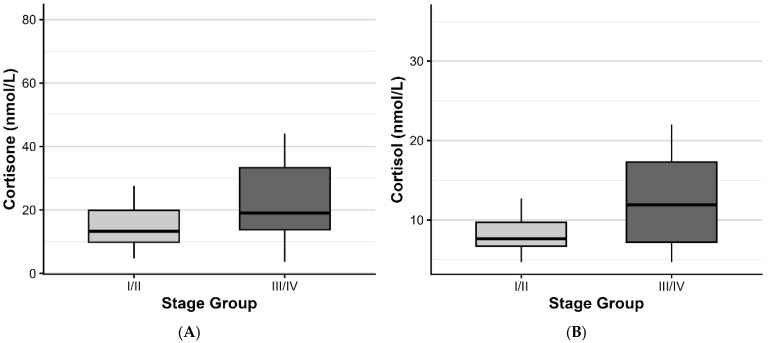
Boxplots showing: (**A**) salivary cortisone and (**B**) cortisol levels in patients with Stage I/II vs. Stage III/IV periodontitis.

Patients with Stage III/IV periodontitis showed significantly higher salivary cortisone and cortisol levels compared with those with Stage I/II disease (Table 1). Median cortisone concentrations increased from 13.30 nmol/L (IQR: 10.00) in Stage I/II to 19.00 nmol/L (IQR: 19.50) in Stage III/IV (*p* = 0.014), corresponding to a medium effect size (Cliff’s δ = 0.35, 95% CI: 0.07–0.58). Similarly, cortisol levels increased from 7.64 nmol/L (IQR: 2.99) to 11.90 nmol/L (IQR: 10.10) (*p* = 0.014; Cliff’s δ = 0.35, 95% CI: 0.06–0.58). Boxplots illustrating these differences are shown in Figure 2.

The cortisol/cortisone ratio did not differ significantly between groups (*p* = 0.436), with a negligible effect size (Cliff’s δ = 0.11, 95% CI: −0.17–0.38), suggesting that although total glucocorticoid levels increased, the balance between active and inactive glucocorticoids remained stable.

### 2.2. Assessment of Potential Confounding Variables

No significant differences were observed between Stage I/II and Stage III/IV groups regarding age (*p* = 0.651), sex distribution (χ^2^ = 0.76, *p* = 0.383), or smoking status (χ^2^ = 0.75, *p* = 0.687). None of these factors were significantly associated with salivary cortisone or cortisol concentrations (all *p* > 0.26).

In multivariable linear regression models adjusting for age, sex, and smoking, Stage III/IV periodontitis remained a significant predictor of both cortisone (β = 0.378, *p* = 0.022) and cortisol (β = 0.346, *p* = 0.004), confirming that the observed elevations in biomarker levels were independent of demographic or behavioral factors.

### 2.3. Correlation Analysis Between Salivary Biomarkers

Cortisone and cortisol were strongly correlated (ρ = 0.523, *p* < 0.001), indicating coordinated activation of the HPA axis. The cortisol/cortisone ratio correlated negatively with cortisone (ρ = −0.631, *p* < 0.001) but showed no significant association with cortisol (ρ = 0.228, *p* = 0.064).

**Figure 3:**
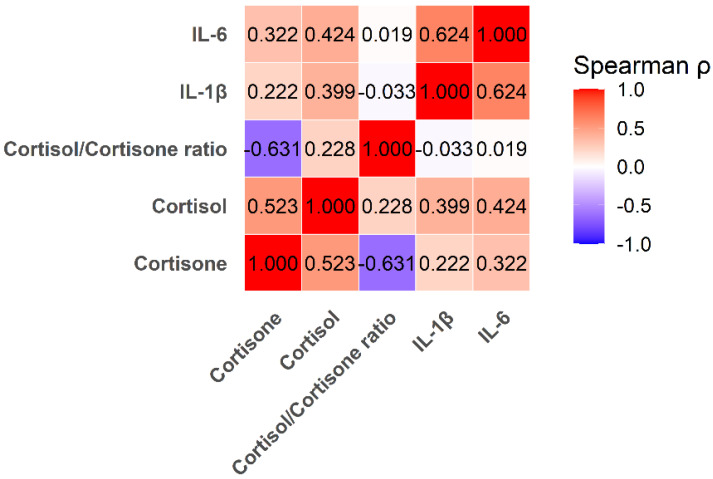
Heatmap showing Spearman correlation coefficients (ρ) among salivary cortisone, cortisol, the cortisol/cortisone ratio, IL-1β, and IL-6. Color intensity reflects the strength and direction of each association (red = positive correlation; blue = negative correlation). Numerical values inside each cell represent Spearman’s ρ calculated from the full sample (*n* = 67).

**Figure 4:**
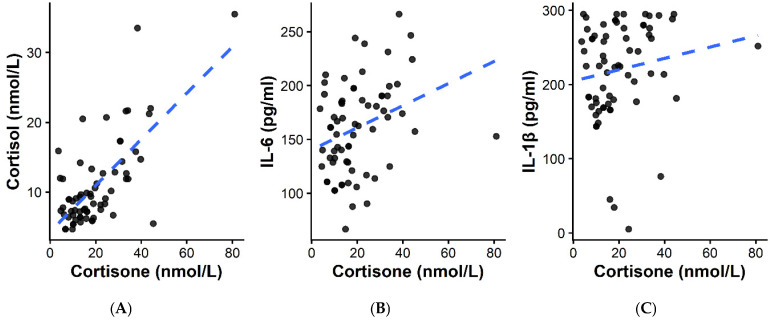
Scatterplots of cortisone vs. cortisol, IL-6, and IL-1β: (**A**) Cortisone vs. cortisol, illustrating a strong positive association consistent with coordinated HPA axis activation (Spearman ρ = 0.523, *p* < 0.001); (**B**) Cortisone vs. IL-6, showing a positive relationship between glucocorticoid levels and systemic inflammation (ρ = 0.322, *p* = 0.008); (**C**) Cortisone vs. IL-1β, demonstrating a weaker but consistent trend (ρ = 0.222, *p* = 0.072).

Both glucocorticoids were positively correlated with inflammatory biomarkers: cortisol showed moderate correlations with IL-1β (ρ = 0.399, *p* < 0.001) and IL-6 (ρ = 0.424, *p* < 0.001), whereas cortisone demonstrated a weaker but statistically significant correlation with IL-6 (ρ = 0.322, *p* = 0.008) and a non-significant trend with IL-1β (ρ = 0.222, *p* = 0.072). IL-1β and IL-6 were strongly correlated (ρ = 0.624, *p* < 0.001). These relationships are illustrated in the correlation heatmap (Figure 3) and cortisone-based scatterplots (Figure 4).

### 2.4. Correlations Between Cortisone and Clinical Periodontal Parameters

Salivary cortisone was significantly associated with several clinical indicators of periodontal severity (Table 2). Higher cortisone levels correlated with increased plaque accumulation (FMPS: ρ = 0.256, *p* = 0.036) and bleeding on probing (BoP: ρ = 0.282, *p* = 0.021). Strong associations were observed with probing depth distribution, including fewer shallow sites (PPD ≤ 3 mm: ρ = −0.331, *p* = 0.006) and more deep pockets (PPD > 5 mm: ρ = 0.421, *p* < 0.001; PPD > 7 mm: ρ = 0.243, *p* = 0.047). Cortisone was inversely related to mild attachment loss (CAL 1–2 mm: ρ = −0.285, *p* = 0.019) and positively related to severe attachment loss (CAL ≥ 5 mm: ρ = 0.332, *p* = 0.006).

A modest correlation was also found for BL/Age (ρ = 0.248, *p* = 0.043), whereas tooth loss showed no association (*p* = 0.774). These findings collectively indicate that elevated cortisone reflects more pronounced periodontal inflammation and tissue destruction.

### 2.5. Diagnostic Performance (ROC Analysis)

**Figure 5:**
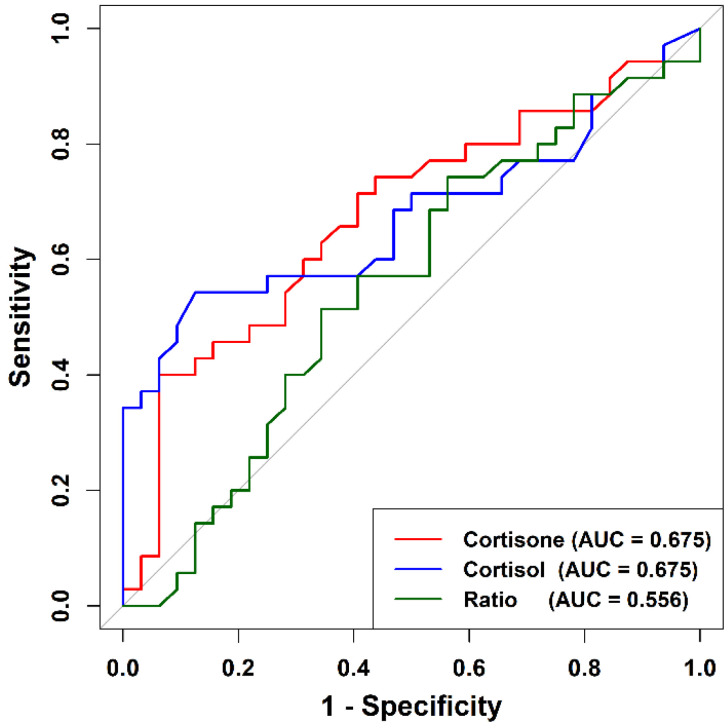
ROC curves for salivary cortisone, cortisol, and the cortisol/cortisone ratio in discriminating Stage III/IV from Stage I/II periodontitis. Cortisone and cortisol showed comparable and moderate discriminatory performance, while the cortisol/cortisone ratio exhibited limited accuracy.

ROC analysis evaluated the ability of salivary biomarkers to discriminate between Stage I/II and Stage III/IV periodontitis (Table 3, Figure 5). Cortisone showed moderate discriminatory performance (AUC = 0.675, 95% CI: 0.543–0.806), with high specificity (93.8%) at an optimal threshold of 28.03 nmol/L and a classification accuracy of 66%. Cortisol demonstrated an identical AUC (0.675, 95% CI: 0.542–0.808) and a slightly higher sensitivity (54%) at a cutoff of 11.57 nmol/L, and an accuracy of 70%.

The cortisol/cortisone ratio exhibited limited discriminatory capacity (AUC = 0.556, 95% CI: 0.414–0.697), with a corresponding accuracy of 60%. Overall, cortisone performed comparably to cortisol and showed strong specificity for identifying advanced periodontitis.

## 3. Discussion

This study provides the first evaluation of salivary cortisone as a biomarker in periodontitis and directly compares its performance with salivary cortisol using LC–MS/MS. The principal findings were that both cortisone and cortisol were significantly elevated in patients with Stage III/IV periodontitis compared with Stage I/II, and that cortisone demonstrated consistent associations with pro-inflammatory cytokine IL-6 and clinical periodontal parameters—FMPS, PPD > 5 mm, CAL ≥ 5 mm, BoP, and BL/Age ratio. These results suggest that cortisone performs similarly to cortisol in reflecting periodontal disease severity and may serve as a reliable alternative biomarker, with a modest advantage of slightly higher specificity in differentiating severe from milder disease.

Both glucocorticoids were significantly higher in patients with advanced periodontitis. The similar magnitude of the stage differences indicates that systemic glucocorticoid activation reflects the increased inflammatory burden characteristic of severe periodontitis. These findings align with previous cortisol-based studies showing elevated glucocorticoid output in periodontitis, including the meta-analysis by Al-Ak’hali et al. [5], the stage-dependent rise reported by Hingorjo et al. [6], and the relationships between cortisol and clinical periodontal severity observed by Antequera et al. [7]. By demonstrating comparable stage discrimination using cortisone, our study adds new evidence that both glucocorticoids respond similarly to periodontal disease severity.

Although cortisone did not provide substantially stronger stage differentiation than cortisol, its slightly higher ROC specificity suggests that it may yield fewer false-positive cases of severe periodontitis. This modest advantage, combined with the known physiological stability of cortisone in saliva, supports its consideration as a complementary or alternative biomarker.

Cortisone demonstrated a significant positive correlation with IL-6, a central cytokine in periodontal pathogenesis and a mediator of systemic inflammatory amplification. IL-6 production increases in response to microbial dysbiosis, immune activation, and tissue destruction [19,20]. Thus, the observed association suggests that cortisone may reflect systemic inflammatory processes associated with periodontitis. A non-significant trend was observed for IL-1β, a cytokine more closely associated with site-specific inflammatory activity [21,22]. This difference is biologically plausible, as single-sample salivary biomarkers tend to better reflect systemic mediators like IL-6 than locally fluctuating cytokines such as IL-1β.

Cortisol showed significant correlations with IL-1β and IL-6 in our dataset. These results were expected and serve primarily as a reference point, since the study’s primary aim was to evaluate cortisone. Taken together, the inflammatory findings indicate that cortisone is biologically responsive to systemic inflammatory activity associated with periodontitis, although effect sizes were moderate.

One of the most meaningful findings of our study is that cortisone demonstrated consistent and significant associations with several key clinical indicators—bleeding on probing, full-mouth plaque score, the distribution of deeper probing depths, and the proportion of sites with CAL ≥ 5 mm. These correlations suggest that systemic glucocorticoid activity may reflect the extent of periodontal inflammation and tissue destruction.

The behavior of cortisone observed in this study is supported by established mechanistic evidence showing that glucocorticoid metabolism is actively involved in periodontal inflammation. Human gingival biopsies demonstrated upregulation of 11β-HSD1 in chronic periodontitis, promoting the intracellular conversion of cortisone to cortisol within inflamed tissues [8]. Animal models showed similar findings—ligature-induced and LPS-induced periodontal inflammation resulted in increased 11β-HSD1 expression, associated with elevated TNF-α, increased osteoclast activity, and accelerated bone loss [9,10]. In periodontal ligament cells, *P. gingivalis* LPS upregulated 11β-HSD1 and drove IL-6, IL-1β, and COX-2 production [11].

Meanwhile, salivary glands predominantly express 11β-HSD2, which converts cortisol to cortisone, generating the primary glucocorticoid metabolite detected in saliva [12]. Human gingival biopsy studies have shown that, in contrast to the marked upregulation of 11β-HSD1 in chronic periodontitis, 11β-HSD2 expression is slightly reduced, resulting in an increased 11β-HSD1/11β-HSD2 ratio within inflamed periodontal tissues [8]. Experimental models of periodontal inflammation support this pattern, indicating that inflammatory conditions do not upregulate 11β-HSD2 and may further contribute to a relative shift toward increased local cortisol levels [9]. The interplay of these two enzyme systems—local activation of cortisol in inflamed tissues and systemic conversion to cortisone in salivary glands—provides an explanation for why cortisone reflects both systemic stress signaling and periodontal destruction. The relatively stable cortisol/cortisone ratio observed from our data is consistent with this dual-system model.

For periodontists, biomarkers that reflect inflammation and periodontal tissue destruction may help improve risk assessment, patient stratification, and monitoring of disease activity. In this study, cortisone showed consistent associations with inflammatory markers and with several key clinical indicators of periodontal severity, suggesting that it may have potential as a salivary biomarker worth further investigation. Although the present findings are encouraging, cortisone cannot yet be considered a validated marker of periodontal disease. Instead, it should be viewed as a promising candidate that warrants additional research in larger, longitudinal studies that also investigate the underlying biological processes involved. As interest in saliva-based diagnostics continues to expand within dentistry [23], cortisone represents a biologically plausible and analytically stable target for future biomarker development efforts.

### 3.1. Study Limitations

This study has several limitations that should be considered when interpreting the findings. First, the cross-sectional design does not allow causal relationships to be established between glucocorticoid levels and periodontal disease progression. Second, the sample size, while adequate for detecting major differences, may have limited the ability to identify weaker associations, particularly for IL-1β. Third, measurements were based on a single morning saliva sample, which reduces but does not completely eliminate diurnal variability. In addition, no microbiological analysis was performed, preventing integration of glucocorticoid responses with microbial profiles.

### 3.2. Future Research Perspectives

Future studies should evaluate salivary cortisone in larger and longitudinal cohorts to clarify its temporal relationship with periodontal disease progression, fluctuations in inflammatory burden, and response to periodontal therapy. Assessing cortisone dynamics before and after treatment may help determine whether changes in glucocorticoid levels reflect improvements in periodontal inflammation or systemic stress responses. Integrating cortisone assessment with microbiological profiling and additional inflammatory and stress-related biomarkers could further elucidate the complex interactions between psychological stress, host immune response, and periodontal tissue destruction.

From a practical and translational perspective, the analytical stability of salivary cortisone and its high specificity for advanced disease suggest potential utility as a research biomarker for patient stratification and risk assessment in studies investigating stress–inflammation interactions in periodontitis. Such applications may be particularly relevant in identifying patients with heightened stress-related endocrine responses. Nevertheless, longitudinal validation, standardization of sampling protocols, and interventional studies are required before any clinical implementation or diagnostic use can be considered.

## 4. Materials and Methods

This cross-sectional study was conducted in accordance with the principles of the Declaration of Helsinki and received approval from the institutional ethics committee (approval number 2285/28 June 2023). All participants provided written informed consent prior to participation.

### 4.1. Study Population

A total of 67 adults diagnosed with periodontitis were recruited consecutively. Diagnosis and staging were performed according to the 2017 EFP/AAP World Workshop criteria [24], enabling classification into Stage I/II or Stage III/IV periodontitis.

#### 4.1.1. Clinical Examination

Comprehensive periodontal examinations were performed by a calibrated periodontist using a UNC-15 periodontal probe. Probing pocket depth (PPD), clinical attachment loss (CAL), bleeding on probing (BoP), full-mouth plaque score (FMPS), and full-mouth bleeding score (FMBS) were recorded. The number of missing teeth due to periodontitis and the bone loss/age (BL/Age) ratio, derived from radiographic assessment of the site with the greatest alveolar bone loss, were also documented.

#### 4.1.2. Inclusion Criteria

Participants were eligible if they were ≥18 years old, diagnosed with periodontitis according to the 2017 EFP/AAP World Workshop case definitions and staging criteria, had a minimum of 15 teeth, and were able to provide written informed consent.

#### 4.1.3. Exclusion Criteria

Participants were excluded if they were pregnant or lactating; had systemic diseases known to affect adrenal or immune function; were diagnosed with endocrine disorders; were receiving corticosteroid, psychotropic, or hormonal medications; had a history of alcohol abuse; or had received antibiotics or anti-inflammatory medication within the preceding three months. Individuals who had undergone periodontal treatment within the previous year were also excluded.

### 4.2. Saliva Collection

Unstimulated whole saliva was collected between 8:00 and 10:00 AM to minimize circadian variation in glucocorticoid secretion. Participants refrained from eating, drinking (except water), smoking, and performing oral hygiene procedures for at least one hour before sampling. Saliva was obtained using the passive drool method, centrifuged to remove cellular debris, and stored at −80 °C until biochemical analysis [25].

### 4.3. Biochemical Assessment

Salivary cortisol and cortisone were measured using liquid chromatography–tandem mass spectrometry (LC–MS/MS) with a commercially available, validated assay (MassChrom^®^ Cortisol/Cortisone in Saliva kit, Chromsystems Instruments & Chemicals GmbH, Munich, Germany). Sample preparation involved protein precipitation followed by LC–MS/MS analysis with stable isotope–labeled internal standards for both analytes, ensuring high analytical specificity.

Calibration was carried out using manufacturer-provided multi-level calibrators covering the physiological concentration range of salivary glucocorticoids. Quality control samples were included in each analytical run and monitored throughout the study. Intra-assay coefficients of variation, reflecting the consistency of repeated measurements within a single analytical run, were <6%, while inter-assay coefficients of variation, indicating reproducibility across different analytical runs, were <10% for both cortisol and cortisone. These values indicate high analytical precision and reproducibility of the measurements.

Analyses were performed according to the manufacturer’s protocol, allowing simultaneous and interference-free measurement of cortisol and cortisone. This approach avoids the cross-reactivity associated with immunoassay-based cortisol measurements and is widely validated for salivary steroid assessment in clinical research [26].

Salivary interleukin-1β (IL-1β) and interleukin-6 (IL-6) were measured using commercially available high-sensitivity enzyme-linked immunosorbent assay (ELISA) kits (BioVendor^®^, Brno, Czech Republic), according to the manufacturer’s instructions, with each sample analyzed in duplicate.

### 4.4. Statistical Analysis

Statistical analyses were performed using R software (version 4.5.1). Normality was assessed using the Shapiro–Wilk test. Because salivary markers were not normally distributed, comparisons between Stage I/II and Stage III/IV periodontitis were conducted using the Mann–Whitney U test, with effect sizes expressed as Cliff’s delta (δ) [27] and 95% confidence intervals. Spearman’s rank correlations were used to evaluate associations among glucocorticoids, cytokines, and clinical periodontal parameters. Multivariable linear regression models were constructed to determine whether periodontitis stage independently predicted salivary cortisone and cortisol levels after adjusting for age, sex, and smoking. Diagnostic performance for distinguishing disease stages was assessed using receiver operating characteristic (ROC) curves, which illustrate the trade-off between sensitivity and specificity across all possible cutoff values, with the area under the curve (AUC) providing a summary measure of overall discriminatory ability [28]. For each salivary biomarker, sensitivity and specificity were calculated across all observed concentration values and plotted as ROC curves. Optimal thresholds were determined using the Youden index by evaluating all ROC-derived cutoff points and selecting the value that provided the best combined sensitivity and specificity [29]. Statistical significance was set at *p* < 0.05.

## 5. Conclusions

This study demonstrated that salivary cortisone and cortisol exhibit comparable patterns in relation to periodontal disease severity. Both glucocorticoids were significantly elevated in Stage III/IV periodontitis and showed coordinated behavior, reflecting activation of the HPA axis in association with periodontal inflammation. Salivary cortisone was significantly associated with IL-6 and showed a consistent positive trend with IL-1β, while salivary cortisol correlated significantly with both inflammatory cytokines. Together, these findings underscore the link between systemic glucocorticoid activity and periodontal inflammatory burden.

In addition, salivary cortisone was associated with multiple clinical indicators of periodontal inflammation and tissue destruction, including a higher proportion of deep probing pocket depths, greater clinical attachment loss, and increased bleeding on probing. Diagnostic performance analysis revealed comparable discriminatory ability for cortisone and cortisol, with cortisone demonstrating higher specificity for identifying advanced stages of periodontitis. Overall, these results suggest that salivary cortisone performs similarly to cortisol and represents a biologically relevant glucocorticoid marker. Further longitudinal and mechanistic studies are warranted to clarify its potential role as an alternative salivary biomarker in periodontal research.

## Figures and Tables

**Table 1:** Comparison of salivary cortisone, cortisol, and cortisol/cortisone ratio between Stage I/II and Stage III/IV periodontitis.

Biomarker	Stage I/II (*n* = 32)	Stage III/IV (*n* = 35)	*p*-Value	Cliff’s δ (95% CI)
Cortisone (nmol/L)	13.30 (10.00)	19.00 (19.50)	0.014 *	0.35 (0.07–0.58)
Cortisol (nmol/L)	7.64 (2.99)	11.90 (10.10)	0.014 *	0.35 (0.06–0.58)
Cortisol/Cortisone ratio	0.55 (0.35)	0.50 (0.25)	0.436	0.11 (−0.17–0.38)

Values are presented as median (interquartile range). Group differences were assessed using the Mann–Whitney U test. Effect sizes are reported as Cliff’s delta (δ) with 95% confidence intervals. Statistically significant *p*-values are indicated by an asterisk (*).

**Table 2:** Correlations between salivary cortisone and clinical periodontal parameters.

Clinical Parameter	Spearman ρ	*p*-Value
FMPS	0.256	0.036 *
FMBS	0.113	0.365
PPD ≤ 3 mm	−0.331	0.006 *
PPD 4–5 mm	0.202	0.101
PPD > 5 mm	0.421	<0.001 *
PPD > 7 mm	0.243	0.047 *
CAL 1–2 mm	−0.285	0.019 *
CAL 3–4 mm	−0.155	0.211
CAL ≥ 5 mm	0.332	0.006 *
BoP	0.282	0.021 *
BL/Age	0.248	0.043 *
Teeth lost	0.036	0.774

Spearman’s rank correlation coefficients (ρ) and corresponding *p*-values are shown. Statistically significant correlations (*p* < 0.05) are indicated with an asterisk (*).

**Table 3:** ROC analysis for salivary cortisone, cortisol, and cortisol/cortisone ratio.

	AUC	95% CI	Optimal Cutoff	Sensitivity (%)	Specificity (%)	Accuracy (%)
Cortisone (nmol/L)	0.675	0.543–0.806	28.03	40	94	66
Cortisol (nmol/L)	0.675	0.542–0.808	11.57	54	88	70
Cortisol/Cortisone ratio	0.556	0.414–0.697	0.659	74	44	60

AUC = area under the ROC curve; 95% CI = 95% confidence interval. Optimal cutoff values were determined using the Youden index, which identifies the threshold that maximizes the combined sensitivity and specificity. Accuracy (%) represents the proportion of correctly classified cases at the selected cutoff.

## Data Availability

Data supporting the findings of this study can be obtained from the corresponding author upon reasonable request, in compliance with ethical and privacy regulations.

## Abbreviations

The following abbreviations are used in this manuscript:

AUC	Area under the curve
ROC	Receiver operating characteristic
CI	Confidence interval
IQR	Interquartile range
CV	Coefficient of variation
HPA	Hypothalamic–pituitary–adrenal
FMPS	Full-mouth plaque score
FMBS	Full-mouth bleeding score
PPD	Probing pocket depth
CAL	Clinical attachment loss
BoP	Bleeding on probing
BL/Age	Bone loss/age ratio
IL-1β	Interleukin-1 beta
IL-6	Interleukin-6
11β-HSD1	11β-hydroxysteroid dehydrogenase type 1
11β-HSD2	11β-hydroxysteroid dehydrogenase type 2
LC–MS/MS	Liquid chromatography–tandem mass spectrometry
ELISA	Enzyme-linked immunosorbent assay
